# Idiopathic membranous nephropathy and IgG4: an interesting relationship 

**DOI:** 10.5414/CN107768

**Published:** 2013-02-05

**Authors:** Edward J. Filippone

**Affiliations:** Division of Nephrology, Department of Medicine, Thomas Jefferson University, Philadelphia, PA, USA

**Keywords:** membranous nephropathy, phospholipase A2 receptor antibodies, IgG4, T-helper cell subsets

## Abstract

Idiopathic membranous nephropathy (iMN) is a single-organ autoimmune disease characterized by subepithelial deposition of immune complexes containing IgG4 resulting in proteinuria, nephrotic syndrome, and, in some, end-stage renal disease. The pathogenesis involves a chronic IgG4 response against specific podocyte antigens which have now been at least partially defined in the neonatal, early childhood, and adult varieties. More has recently been learned about the genetic predisposition as well. This review discusses the pathophysiology of iMN in light of these discoveries and what is known about the genesis and potential clinical ramifications of an antigen-specific IgG4 response.

## Introduction 

Membranous nephropathy (MN) is one of the most common causes of nephrotic syndrome in adults and is the second commonest glomerulopathy to progress to end-stage renal disease (ESRD). Most cases are idiopathic (iMN), but ~ 20% are secondary to various causes, including cancers, infections, autoimmune diseases, and medications [1]. The characteristic feature is immune complex (IC) formation/deposition on the sub-epithelial side of the glomerular basement membrane (GBM) resulting in activation of complement, oxidative stress, podocyte dysfunction, proteinuria, and, in some, progressive loss of renal function [2]. These ICs contain immunoglobulin (Ig), specifically IgG. Complement components, usually C3, may also be found. 

It is well established that an IgG4 response is intimately involved in the pathogenesis of iMN. It has been shown repeatedly that IgG4 predominates in the glomerular ICs in iMN, less so in secondary cases. Bannister et al. [3] found staining for IgG4 in 100% of 10 patients with iMN, although IgG3 stained more intensely. Doi et al. [4] found IgG4 deposits in 100% of 12 patients with iMN; additional weak IgG1 staining was found in 7. Haas [5] found IgG4 deposits in 100% of 28 patients with apparent iMN. Overall, IgG4 staining was the strongest, but IgG1 was found in 100%, IgG2 in 79%, and IgG3 in 75%. In 6 of these cases, IgG3 staining was approximately equal to IgG4. Kuroki et al. [6] found IgG4 in 100%, IgG1 in 81%, and IgG2 and IgG3 in 25% each, in 16 patients with iMN; IgG4 had the most intense reaction. Noel et al. [7] studied 16 patients with iMN and found IgG4 deposits in 81% and IgG1 in 75%. In 21 patients with iMN, Imai et al. [8] found the strongest deposition for IgG4 compared to other subclasses and to other glomerulopathies; the percentage of patients who were positive was unspecified. 

In the setting of secondary MN, other subclasses are usually found to predominate. In lupus MN, some have reported IgG4 [8, 9], but in the majority of cases this subclass can not be found [10]. Ohtani et al. [11] compared subclass distribution between 15 patients with iMN and 10 with malignancy-associated MN. There was no difference in intensity of staining for IgG4 between groups, but IgG1 and IgG2 staining was significantly stronger in the malignancy group. A more recent study found negative staining in 7 of 8 malignancy-associated cases and suggested that a negative stain for IgG4 in suspected iMN should prompt a search for underlying malignancy [12]. In a series of 26 patients with a monoclonal immune complex glomerular disease, 14 had MN and 12 had membranoproliferative glomerulonephritis (GN) [13]. Subclass analysis of 11 of the patients with MN showed no IgG4; 7 of the 11 had IgG1 and 2 each had IgG2 and IgG3. Finally, MN may be found in renal transplants, either as a recurrent disease or arising de novo. IgG4 has been found to predominate in recurrent cases of MN, but not in those considered de novo. In one series of 11 patients, all 7 cases of recurrent MN stained for IgG4 (dominant or co-dominant); however, in the 4 cases of de novo or atypical MN, 3 showed dominant IgG1 and the fourth co-dominant IgG1 and IgG4 [13]. 

The exact pathophysiology of iMN has remained an enigma. Experimentally, a similar disease can be produced in rats (Heymann nephritis) via antibodies directed primarily against megalin, a protein expressed on the epithelial surface in clathrin-coated pits on the soles of podocyte foot processes [15, 16]. This disease can be produced actively in rats by immunization with various preparations such as Fx1A, a rat proximal tubular extract, or passively by administration of serum raised by similar immunizations in rabbits. Unfortunately, this does not apply to humans who do not express megalin in podocytes. The antigenic target in human iMN was previously unknown, but much has been learned recently. 

## Target antigens 

In human neonatal MN, pathogenic IgG4 and IgG1 antibodies against neutral endopeptidase can be detected in maternal serum from mothers deficient in this enzyme who were presumably immunized during prior pregnancies [17, 18]. These antibodies cross the placenta and result in alloimmune MN in the neonatal period. This phenomenon has been detected in at least three families. The oldest patient (age 20) developed severe renal impairment. 

In early childhood iMN, unusually cationic bovine serum albumin (BSA), along with BSA-specific IgG4 and IgG1, were detected in serum, and co-localized in subepithelial IC deposits in 4 of 5 consecutive patients who were less than 5 years of age [19]. In the same series, 7 of 41 adults also had high titers of the antibodies, but the circulating BSA was neutral or anionic, and this antigen-antibody complex was not involved in the pathogenesis of their disease. 

In adults with iMN, IgG4 antibodies against a variety of proteins can now be detected in the serum of a significant proportion of patients. They can also be eluted from glomeruli and co-localized along with IgG4 within the ICs on the subepithelial side of the GBM. The first to be described were antibodies against the M-type phospholipase A2 receptor (PLA2R), which is normally expressed on the cell membrane of podocytes. In their seminal paper, Beck et al. [20] detected these IgG4 antibodies in 26 of 37 American patients with iMN. The method involved western blotting of patient serum under non-reducing conditions against extracts of both normal human glomeruli (HGE) as well as recombinant human PLA2R expressed in a transfected human embryonic kidney (HEK) cell line. After removal of IgG, a 185 kD protein band was found to be the reactive component and was shown to correspond to the PLA2R. Neither circulating PLA2R nor ICs containing it could be detected in the circulation. Furthermore, PLA2R and IgG4 co-localized in the immune deposits in patients with iMN, but not in those with a secondary cause. 

Utilizing the same methodology in 60 Chinese patients with iMN, anti-PLA2R antibody was found in 49 (81.7%) [21]. By enhancing the sensitivity of the assay (less dilution of serum, increased concentration of detection antibody, increased exposure time), an additional 10 patients were found to be positive (59 of 60 were positive overall). In comparison, only 1 of 20 patients with membranous lupus was positive, even with the more sensitive methodology. Of 16 patients with hepatitis B associated MN, I was positive with the standard assay, 2 more with the sensitive assay. Of 10 patients with malignancy associated MN, 3 were positive with the standard assay, none additional with the more sensitive one. In all positive cases, the subclass was found to be IgG4. In another study of a cohort of 18 Dutch patients, 14 had detectable antibodies [22]. 

These findings have been replicated by other groups. Debiec and Ronco [23] studied 42 French patients using both western blotting of normal HGE and indirect immunofluorescence (IIF) of transfected HEK cells. They found serum reactivity in 24 (57%); 31 patients had PLA2R detected in immune deposits by confocal microscopy. Utilizing an IIF assay also against transfected HEK cells, circulating antibody was detected in 52% of 100 German patients with iMN; it was not found in healthy controls, secondary MN, or other glomerulopathies [24]. 

In the transplant setting, Debiec et al. [25] studied 10 patients with recurrent MN and 9 with de novo MN. Five of the 10 patients with recurrence had both anti-PLA2R antibodies detectable in the circulation and PLA2R antigen in immune deposits; none of 9 with de novo MN had either of these findings. Notably, of 6 patients whose original disease was iMN and with no evidence of recurrence, 3 had high titers of anti-PLA2R antibodies at the time of transplantation. 

Beck et al. [26] evaluated at the utility of following serial levels of anti-PLA2R antibodies as a guide to therapy. They retrospectively studied 35 patients from 2 cohorts who were treated with the B-cell depleting antibody rituximab. Pretreatment samples from 25 of 35 (71%) contained detectable antibodies by their western blot methodology. Of the 17 (68%) patients with declining/disappearing levels by 12 months, 88% attained remission by 2 years; change in antibody level preceded change in proteinuria. This compares to a 33% remission rate in those with persisting levels and a 78% rate in those with negative levels pretreatment. 

Others have detected IgG4 antibodies against different antigens in the serum and in renal tissue of patients with iMN. Prunotto et al. demonstrated a de novo expression of aldose reductase (AR) and superoxide dismutase-2 (SOD) in glomeruli of patients with iMN as compared to normal glomeruli and other glomerulopathies [27]. Both antigens were found in IC deposits and co-localized with IgG4 and complement in these patients, and IgG4 eluted from glomeruli was specific for them. Furthermore, high titer circulating IgG4 against these antigens was found in over 50% of patients as well. Bruschi et al. [28] found circulating IgG4 antibodies against α-enolase in the serum of 25% of 131 iMN patients. Confocal- and immuno electron-microscopy showed co-localization of α-enolase with IgG4 and C5b-9 in immune deposits. Similar to AR and SOD, α-enolase is not expressed normally on the cell membrane of podocytes, but can be found there in iMN patients. 

## Genes and membranous nephropathy 

A genetic predisposition has been known to underlie iMN for many years. In 1979, Klouda et al. [29] found a strong association in Caucasian iMN patients with the HLA-DR3 antigen. HLA-B8 and B18, in linkage disequilibrium with DR3, were also increased. The F1 allele of properdin factor B (BfF1) associates with both HLA-B18 and HLA-DR3 [30]. This B18-BfF1-DR3 haplotype was strongly associated with iMN in an English population. Muller et al. [31] in 21 German patients, confirmed the association with HLA-DR3 and noted a strong association with the MT-2 supertypic specificity; however, they found an association with neither HLA-B18 nor the B18-BfF1-DR3 haplotype. On the other hand, in Japanese patients with iMN, there is a strong association with the HLA-DR2 and MT-1 antigens [32, 33]. 

More recently, Stanescu et al. [34] performed a genomewide association study of single nucleotide polymorphisms (SNPs) in 556 Caucasian patients with iMN. The SNP rs2187668 for HLA-DQA1 strongly associated with the disease, with an odds ratio (OR) of 20, as did the intronic SNP rs4664308 for the PLA2R (OR of 4); homozygosity for both at-risk SNPs markedly increased the risk (OR of 78.5). Two other groups studied SNPs resulting in amino acid substitutions in the PLA2R protein in Asian patients with iMN. Both groups found significant associations at rs35771982, a locus different from the one detected in Caucasians. Liu et al. [35] studied 129 Chinese and found a significant association with the G allele (OR 1.9 vs. C allele). On the other hand, Kim et al. [36] found a significant association with the C allele at this same locus with an adjusted OR of 2.934 for homozygosity (vs. CG or GG genotypes) in 199 Korean patients. 

Polymorphisms at other loci have also been associated with risk for iMN. These include the genes for nephrin [37], the inflammatory mediators IL-6 [38] and tumor necrosis factor-α [39], and plasminogen activator inhibitor-1 [40]. Hence the risk for developing iMN involves SNPs at numerous genes in addition to those for HLA antigens and the PLA2R. 

## T cell subsets in membranous nephropathy 

A T-helper (Th)-2 polarization is said to underlie iMN [41, 42, 43]. Naïve T-helper (Th0) cells can differentiate into various subsets after antigen recognition based on the prevailing cytokine milieu [44]. These subsets then produce select cytokine profiles and have somewhat specific roles in immune system function, although overlap as well as plasticity occurs. They include Th1 cells (involved in delayed-type hypersensitivity and IgG1 antibody production), Th2 cells (fostering IgE and IgG4 antibody production and causing allergy), Th17 cells (promoting inflammation) and regulatory T cells (Tregs, regulating the activity of other effector cell populations). After antigenic presentation, Th0 cells would be driven towards a Th1 phenotype by the presence of IL-12; a Th2 phenotype by IL-4; a Th17 phenotype by TGF-β plus IL-6, IL-21, and IL-23; and towards production of Tregs by TGF-β in the absence of associated inflammatory cytokines (such as IL6). 

Once differentiated, Th1 cells produce interferon-γ (Inf-γ) and interleukin (IL)-2 while Th2 cytokines include IL-4, Il-5, IL-9, and IL-13. Th17 cells produce IL-17, IL-21, and IL-22, while Tregs are notable for their production of IL-10 and transforming growth factor (TGF)-β. 

Evidence of Th polarization has been studied in patients with iMN by several groups. Hirayama et al. [41] used flow cytometry to study cytokine expression in the CD4+ cells of 8 patients. IL-2+ cells (Th1) were lower than normal, Inf-γ (Th1) and IL-4 (Th2) were the same, but IL-10 (Treg) cells were higher. Using the same methodology, Masutani et al. [42] also showed no difference in Inf-γ+ cells but did find increased IL-4+ cells in 24 iMN patients. This resulted in a lower Th1/Th2 ratio (~ 5) compared to normals (~ 8) and other nephrotic illnesses (~ 10), suggesting a Th2 predominance. Kuroki et al. [43] showed increased mRNA from peripheral blood mononuclear cells for the Treg cytokine IL-10 compared to normal, although no difference for Il-4 or the Th1 cytokine Inf-γ. However, a greater than normal IgG4 response was produced from B-cell cultures when exposed to IL-4, a Th2 cytokine, but not Il-10, IL-13, or Inf-γ. Total circulating IgG4 levels were in the normal range. Together these studies support a modified Th2 response underlying iMN; that is, a Th2 polarization modified by activation of a Treg response producing antigen specific IgG4 antibodies. 

## IgG4: Biology 

IgG4 is a unique subclass of IgG [45, 46]. It was the last to be discovered and exists in the lowest concentration of all 4 subclasses (~ 4% of total IgG). Due to relatively weak inter-chain binding with easy susceptibility to reduction, it is continuously involved in half molecule exchanges in vivo known as “Fab arm exchange” [47]. The result is asymmetric antibodies with two different antigenic specificities. Thus, although structurally hetero-divalent, IgG4 behaves as a functionally monovalent, non cross-linking antibody incapable of producing large immune complexes. It does not bind C1q and cannot activate complement via the classical pathway [48]. Alternative pathway activation may be possible. There is only weak binding to certain FcgRs (none to FcgRIIa or FcgRIIIb, only weakly to FcgRI) resulting in reduced capacity to activate other immune effector cells [49]. 

It is also possible that IgG4-containing ICs activate complement via the mannose-binding lectin (MBL) pathway. This pathway is reportedly involved in various immune complex glomerulopathies [50], most notably IgA nephropathy (IgAN) [51]. MBL has been detected in the glomerular immune deposits of iMN by several groups [50, 52]. It has been hypothesized that hypoglycosylated anti-PLA2R IgG4 results in complement activation via MBL [53, 54]. This is somewhat analogous to MBL activation by the hypogalactosylated IgA1 of IgAN. 

The regulation of IgG4 production is somewhat similar to IgE. It requires typical Th2 cytokines (IL-4 or IL-13) for appropriate class switch [55]. Hence, antigens associated with an IgE response, such as allergens and parasitic infestations, are also good inducers of IgG4. Subsequent exposure to IL-10 produced by Tregs can then drive production, and, depending on the timing, can down regulate IgE [56, 57]. Thus, IgG4 can be produced without an associated IgE response, the so-called “modified Th2 response.” 

Such a modified Th2 response is not uncommon and may occur in normal individuals. It can be found in situations with chronic antigenic exposure, e.g., in bee keepers and those occupationally exposed to animal proteins [45]. It may be viewed as a healthy response to an innocuous antigen. An IgG4 response is slow to develop, usually taking many months of antigenic exposure before becoming prominent. In many such situations IgG4 behaves merely as an innocent bystander. 

## IgG4: Clinical effects 

The clinical manifestations of an antigen specific IgG4 reaction are thus quite variable (Table 1). There may be no detectable clinical manifestations at all, as noted above in beekeepers. Sometimes an IgG4 response is anti-inflammatory, mitigating the effects of pathologic IgE and IgG responses. Examples include allergen specific immunotherapy (sIT) and chronic helminthic infections. During sIT, as the IgG4 level rises, symptoms abate [55, 57]. This is at least in part due to competitive binding of the allergen so that IgE receptors on effector cells such as mast cells and basophils cannot be activated. Other times, however, an IgG4 response is clearly associated with disease or produces unwanted clinical effects. 

In both pemphigus vulgaris and foliaceus, IgG4 antibodies directly produce acantholysis, independent of complement activation or FcR binding [58]. In bullous pemphigoid, pathogenic IgG4 antibodies attack the dermal epidermal junction. In active pemphigus vulgaris, IgG4 antibodies against desmogleins predominate, while in bullous pemphigoid the IgG4 reaction is directed against epitopes in type XVII collagen. In these skin diseases, the IgG4 antibodies are not simply associated phenomena but are on the causal pathway of the disease. 

During chronic administration of therapeutic proteins, an IgG4 response may unfortunately abrogate their therapeutic benefits. Examples include an IgG4 response inhibiting the effect of Factor VIII infusions in hemophilia [59] and anti-chimeric IgG4 antibodies inhibiting the effects of infliximab in rheumatoid arthritis patients [60]. 

IgG4-related systemic disease is a recently named multisystem disease, originally described as autoimmune pancreatitis (AIP) [56, 57]. Usually involving elderly men, features include elevated total IgG and IgG4, elevated IgE, hypocomplementemia, and eosinophilia. Histologically, there is a lymphoplasmacytic infiltrate with > 10 IgG4+ plasma cells (proposed cut-offs vary from > 10 to > 50/hpf) and an elevated IgG4+/total IgG+ plasma cell ratio (≥ 0.4). Fibrosis usually coexists, frequently in a swirling pattern. Although almost any organ system can be involved, common manifestations besides AIP include cholangitis, sialadeniis, retroperitoneal fibrosis, aortitis, lymphadenopathy, pneumonitis, tubulointerstitial nephritis, and prostatitis, among others. This disease is characteristically steroid sensitive, although relapses may occur. Some patients satisfy a classic Th2 paradigm (both IgE and IgG4 elevated), while others are more like a modified Th2 response (normal levels of IgE, elevated IgG4). It remains unproven that IgG4 is directly pathogenic in this entity. In fact, it could be postulated that the IgG4 response is otherwise dampening what would have been a more aggressive immune response. 

Renal involvement is common in IgG4-RSD, detectable radiologically in 30% of cases of AIP in the form of diffuse patchy cortical involvement or hypo-attenuating round or wedge shaped lesions [63]. Perhaps 20% to more than 40% of those with suspected IgG4-RSD may have tubulointerstitial nephritis (IgG4-TIN) with elevated IgG4+ plasma cells (IgG4+ PCs, proposed diagnostic cut-offs of > 10/hpf with an IgG4+ PC/total IgG+ PC ratio ≥ 0.4), variable degrees of fibrosis, and frequently tubular basement membrane deposits [62, 64, 65]. A small percentage of IgG4-TIN cases have co-existing MN [64, 66, 67, 68]. In these cases, IgG4 is the predominant subclass in the glomerular immune complexes, as in iMN. How the pathogenesis of MN in the setting of IgG4-TIN compares to that of iMN is unknown; it remains unclear if the same antigens (e.g., PLA2R, AR, etc.) on podocytes are involved as in iMN, although in 1 case circulating anti-PLA2R antibodies were not detectable [67]. 

Although the presence of > 10 IgG4+ PCs/hpf and/or an elevated ratio is required to diagnose IgG4-TIN, it is clearly not specific. Nearly one third of cases of pauci-immune GN had this level of IgG4+PCs in one series [65]. In another series of 100 cases of TIN (primary or associated with various glomerulopathies), 11 had > 10 IgG4+ PCs/HPF [69]. These included 5 of 16 with necrotizing and crescentic GN, 2 of 8 with diabetic nephropathy, and 2 of 12 with idiopathic TIN. In a study of 44 patients with primary TIN, 12 had ≥ 10 IgG4+ PCs/HPF and 25 had at least 1/HPF [70]. We have recently reported IgG4+ PCs involved in interstitial inflammation in a significant percentage (over 50%) of cases of MN caused by SLE, with 2 of 21 patients having > 10 IgG4+ PCs/hpf [10]. Hence, the mere presence of these cells in a TIN is neither diagnostic nor specific, but must be interpreted in the context of the overall clinical picture. 

Other GNs in addition to iMN involve an IgG4 response. These include anti-neutrophil cytoplasmic antibody (ANCA) positive necrotizing and crescentic GN, circulating anti-glomerular basement membrane mediated GN, and fibrillary GN. In granulomatous polyangiitis (previously called Wegener’s) anti-proteinase-3 ANCA may be predominantly IgG4 in some, but not all, patients [71]. This is in addition to the interstitial IgG4+ PCs noted above. IgG4 (along with IgG2) naturally occurring anti-GBM antibodies were found in the sera of 100% (5 of 5) of normal blood donors in China [72]. The same group found such antibodies in 59% of 73 Chinese patients with anti-GBM disease, although the frequency of IgG1 and IgG3 increased with increasing severity of initial renal impairment [73]. This has been noted in other reports as well. In a series of 11 French patients with anti-GBM antibody mediated GN (3 with concurrent pulmonary disease and hence Goodpasture’s syndrome), 8 had predominantly IgG4 antibodies [7]. In a series of 9 German patients with anti-GBM disease, however, IgG1 was the predominant isotype (75%), with IgG4 minimally detected (2.2%) [74]. Iskandar et al. reported a series of 28 patients with fibrillary GN [75]. All 13 patients with IgG subclass determination had predominant IgG4; in 11 of the 13 it was the sole subclass detected. In another series of 19 patients with fibrillary GN, 17 had IgG4 detected; the other 2 had only IgG1 [76]. 

## Conclusion 

While much has been learned recently regarding the pathogenesis of iMN, many questions still remain. It clearly represents a single organ autoimmune disease. A genetic basis for risk exists but it remains to be determined what the most relevant factors are and how to apply these data in the clinic. Target antigen(s) on podocytes have now been defined, and antibodies against one or more of these antigens can be detected in the circulation of the majority of patients. That the vast majority of these antibodies are of the IgG4 subclass has been clearly established. It remains unknown, however, what causes the chronic antigenic presentation required to initiate a modified Th2 response with IgG4 predominance. Is PLA2R the initiating antigen? What initiates the disease in those patients with no detectable anti-PLA2R antibodies? What is the role of neo-expressed antigens such as AR and SOD? Are there other, as yet undefined, antigens of equal or greater importance? Why do some patients with high levels of anti-PLA2R antibodies at the time of transplantation not have recurrence? Can the presence and/or serial titers of podocyte specific antibodies (e.g., anti-PLA2R) be used to guide immunosuppression? Hopefully, as more knowledge accumulates, we may be better able to tailor therapy for the individual patient. 

**Table 1.** Clinical Manifestations of an IgG4 response.

None
Examples: Beekeepers, animal husbandry workers
Mitigation of an otherwise more inflammatory response
Allergen-specific immunotherapy
Parasitic infestations
Abrogation of therapeutic benefit
Factor VIII infusions
Infliximab therapy
Disease-associated
IgG4-related systemic disease
Fibrillary glomerulonephritis
Disease causing
Pemphigus
Bullous pemphigoid
Idiopathic membranous nephropathy
Anti-GBM antibody disease (some cases)
Anti-neutrophil cytoplasmic antibody positive GN (some cases)
